# Clinical characteristics and prognosis in systemic lupus erythematosus-associated pulmonary arterial hypertension based on consensus clustering and risk prediction model

**DOI:** 10.1186/s13075-023-03139-y

**Published:** 2023-08-23

**Authors:** Mengmeng Dai, Chunyi Zhang, Chaoying Li, Qianqian Wang, Congcong Gao, Runzhi Yue, Menghui Yao, Zhaohui Su, Zhaohui Zheng

**Affiliations:** Department of Rheumatology, The First Affiliated Hospital of Zhengzhou University, Zhengzhou, China

**Keywords:** Systemic lupus erythematosus, Pulmonary arterial hypertension, Consensus clustering, Risk prediction model, Prognosis

## Abstract

**Background:**

Pulmonary arterial hypertension (PAH) is a severe complication of systemic lupus erythematosus (SLE). This study aims to explore the clinical characteristics and prognosis in SLE-PAH based on consensus clustering and risk prediction model.

**Methods:**

A total of 205 PAH (including 163 SLE-PAH and 42 idiopathic PAH) patients were enrolled retrospectively based on medical records at the First Affiliated Hospital of Zhengzhou University from July 2014 to June 2021. Unsupervised consensus clustering was used to identify SLE-PAH subtypes that best represent the data pattern. The Kaplan–Meier survival was analyzed in different subtypes. Besides, the least absolute shrinkage and selection operator combined with Cox proportional hazards regression model were performed to construct the SLE-PAH risk prediction model.

**Results:**

Clustering analysis defined two subtypes, cluster 1 (*n* = 134) and cluster 2 (*n* = 29). Compared with cluster 1, SLE-PAH patients in cluster 2 had less favorable levels of poor cardiac, kidney, and coagulation function markers, with higher SLE disease activity, less frequency of PAH medications, and lower survival rate within 2 years (86.2% vs. 92.8%) (*P* < 0.05). The risk prediction model was also constructed, including older age at diagnosis (≥ 38 years), anti-dsDNA antibody, neuropsychiatric lupus, and platelet distribution width (PDW).

**Conclusions:**

Consensus clustering identified two distinct SLE-PAH subtypes which were associated with survival outcomes. Four prognostic factors for death were discovered to construct the SLE-PAH risk prediction model.

**Supplementary Information:**

The online version contains supplementary material available at 10.1186/s13075-023-03139-y.

## Introduction

Pulmonary arterial hypertension (PAH) is a devastating complication of systemic lupus erythematosus (SLE) [1]. Compared to Western countries, SLE-PAH occurs more frequently in East Asia, carrying high mortality and morbidity [2, 3]. The 5-year overall survival rate varies from 68 to 84% [4, 5]. Although PAH-targeted drugs (PTDs) have been widely prescribed over decades, a fraction of SLE-PAH patients still have poor responses to therapy [6]. This suggests that unique clinical characteristics and immune microenvironment dysregulation in SLE could not be ignored. Apart from cardiopulmonary injury, SLE-PAH patients may also suffer from other organ involvements, implicating the kidney, skin, and central nervous system [7]. These multiple clinical phenotypes could impact their treatment response and prognosis. Hence, recognizing subtypes of SLE-PAH would help to tailor individualized regimens and improve patients’ outcomes [8].

Up to now, a variety of unsupervised clustering methods have been used for subtyping diseases, such as hierarchical clustering, k-means, and latent class analysis [9–11]. In 2018, Sun et al. classified SLE-PAH into two subtypes based on k-means, namely the vasculitic subtype and vasculopathic subtype, attempting to interpret the inflammatory and non-inflammatory pathogenesis of SLE-PAH [11]. Nevertheless, any single clustering method cannot avoid result bias, and its rationality and reliability lack unified standard verification [12]. Consensus clustering, an unsupervised integrative clustering methodology, comes into the spotlight. It can aggregate multiple clustering into a more stable clustering via resampling, which has been introduced in many studies [13–15]. For instance, by consensus clustering, Zheng et al. identified three clusters of chronic kidney disease patients and found a strong association between clusters and different clinical outcomes [15]. Therefore, re-classifying SLE-PAH by an integrative clustering analysis and exploring its clinical characteristics and outcomes would be clinically informative.

Currently, several risk stratification systems of pulmonary hypertension (PH) have been proposed, such as comprehensive and simplified risk assessment from the 2022 European Society of Cardiology (ESC)/European Respiratory Society (ERS) PH guidelines [16], or PAH risk score calculators released by Registry to Evaluate Early and Long-Term PAH Disease Management (REVEAL) [17, 18]. Conversely, due to pleiotropic clinical phenotypes in SLE-PAH patients, some risk profile tools are not good at discriminating high-risk patients, as well as partial variables that are invasive, time-consuming, and costly [19]. Thus, an accurate, non-invasive, and economical SLE-PAH risk prediction model is also needed.

**Fig. 1:**
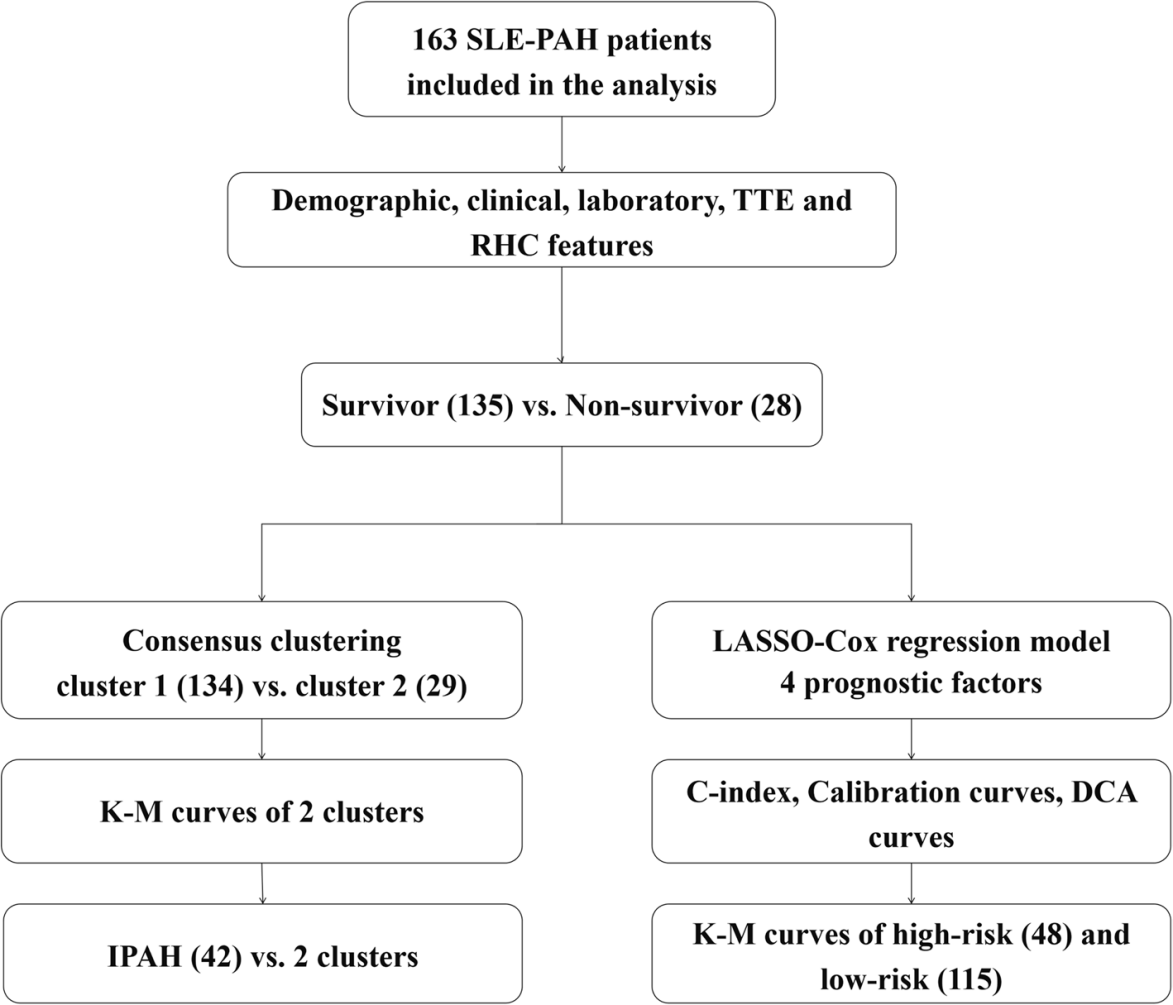
The flow chart of this study. SLE, systemic lupus erythematosus; PAH, pulmonary arterial hypertension; TTE, transthoracic echocardiography; RHC, right heart catheterization; K-M, Kaplan–Meier; IPAH, idiopathic PAH, LASSO, the least absolute shrinkage and selection operator; DCA, decision curve analysis

In general, this study aimed to classify SLE-PAH based on consensus clustering analysis and construct a risk prediction model, for gaining a bettering understanding of SLE-PAH clinical characteristics, treatment, and outcomes. The flow chart of this study was shown in Fig. 1.

## Methods

### Patient selection

Patients diagnosed with SLE-PAH and idiopathic PAH (IPAH) in the First Affiliated Hospital of Zhengzhou University between July 2014 and June 2021 were identified in this retrospective study. SLE patients fulfilled the 1997 updated American College of Rheumatology (ACR) criteria or the Systemic Lupus International Collaborating Clinics (SLICC) group 2012 revised SLE classification criteria [20, 21]. PAH was defined as mean pulmonary artery pressure (mPAP) ≥ 20 mmHg at rest, pulmonary arterial wedge pressure ≤ 15 mmHg, and pulmonary vascular resistance (PVR) > 2 Wood units by right heart catheterization (RHC) [16] or a two consecutive pulmonary artery systolic pressure (PASP) values ≥ 40 mmHg within 3 months by transthoracic echocardiography (TTE) [22].

Patients aged < 16 years or those with a history of recent blood transfusion, recent pregnancy, congenital heart disease, rheumatic heart disease, hypertensive heart disease, myocardial infarction, pulmonary venous occlusion, pulmonary embolism, portal hypertension, chronic obstructive pulmonary disease, pulmonary malignancy, schistosomiasis, left heart diseases, or other connective tissue diseases were excluded.

### Collection of clinical data

The date of baseline was defined as the date of SLE-PAH/IPAH diagnosis confirmed by RHC or TTE. Disease duration was defined as the time from symptom onset to SLE-PAH/IPAH diagnosis. Demographic information, clinical features, laboratory findings, and RHC and TTE parameters at the time of diagnosis were collected from hospital records. Anti-dsDNA antibody was detected by indirect immunofluorescence. The severity of SLE was evaluated by the systemic lupus erythematosus disease activity index-2000 (SLEDAI-2 K) [23]. Patients had planned and recorded comprehensive follow-up evaluations every 3 to 12 months. To be included, patients had to have been followed up for at least 1 year. The endpoint was death from any cause. The follow-up time to endpoint was calculated from the date of SLE-PAH/IPAH diagnosis to the date of death from any cause or to the date of last follow-up (up to June 30, 2022).

### Statistical analysis

Statistical analysis was performed using SPSS 26.0 software (IBM), R software version 4.2.2 (The R Foundation for Statistical Computing), and Prism 9.3.1 software (GraphPad Software). Continuous variables were described as median (interquartile range [IQR]) and compared using the Mann–Whitney *U* test or Kruskal–Wallis test. Bonferroni was used to correct the *P* value for the post hoc test. Categorical variables were expressed as frequency (percentage) and compared using a chi-square test. Consensus clustering analysis was performed using the “ConsensusClusterPlus” R package [24]. The optimal number of clusters was determined by the cumulative distribution function (CDF) curves, consensus clustering score, and consensus plots. Principal component analysis (PCA) was conducted to display the geometrical distance of different subtypes. Kaplan–Meier (K-M) method was applied to describe the survival fractions, the log-rank test was conducted to compare overall survival distributions, and the weighted K-M test was used to compare the short-term survival difference using the R package “ComparisonSurv.”

As for the construction of the risk prediction model, SLE-PAH variables were preliminarily selected based on expert opinion and previous literature. All continuous variables were tested for linear trend, and which do not satisfy linearity would be converted to categorical variables. The cutoff value of these categorical variables was determined by clinical significance, or survival ROC function using the R package “survivalROC.” Subsequently, LASSO regression and univariate Cox analysis were performed to further examine prognostic variables using the R package “glmnet” [25]. Prognostic variables with *P* < 0.1 were then considered for multivariable modeling, before checking that the proportionality of the hazards assumption was met (Supplementary Table S1). The risk prediction model was built using the R package “rms,” and the bootstrap approach was used to validate the model internally. In addition, the C-index, calibration curves, and decision curve analysis (DCA) curves were also conducted for evaluating the model’s discrimination, calibration, and clinical practicability, respectively. Finally, the cohort was divided into high-risk or low-risk groups based on the cutoff value of the risk score, and survival distributions were estimated by the K-M method. Statistical significance was considered *P* < 0.05.

## Results

### SLE-PAH patient characteristics

A total of 163 patients with SLE-PAH were enrolled in this study, including 96.2% (157/163) females, with a median age at diagnosis at 37.0 years (IQR 30.0–49.0), ranging from 16 to 81 years, and the median disease duration was 24 months (IQR 3.0–84.0). All SLE-PAH patients underwent TTE and 46 underwent RHC examination. Besides, there were 100 (61.1%) patients complicated with serositis, 69 (42.6%) with lupus nephritis, and 43 (26.4%) with cardiac disorder. One hundred thirteen (69.3%) patients were in WHO Function Class (WHO FC) III–IV, and the median 6-min walk distance (6MWD) was 436.0 m (IQR 282.0, 534.0). Immunological variables revealed that 160 (98.1%) participants had anti-nuclear antibody (ANA) positivity, followed by 110 (67.9%) anti-Ro52 antibody positivity, 105 (64.8%) anti-nRNP/Sm antibody positivity, and 95 (59.4%) anti-SSA antibody positivity. A median mPAP in RHC was 40.0 mmHg (IQR 28.8, 49.3). Furthermore, 153 (93.9%) patients received glucocorticoids and 99 (60.7%) were treated with immunosuppressants, encompassing 43 (26.4%) mycophenolate mofetil (MMF) and 39 (23.9%) cyclophosphamide (CYC). Besides, 93 (57.1%) patients took PTDs treatment, comprising 55 (33.7%) phosphodiesterase-5 inhibitors (PDE-5Is), 65 (39.9%) endothelin receptor antagonists (ERAs), and 18 (11.1%) prostacyclin (PGI2). Of note, monotherapy of PTDs occupied 68 (41.7%) and combination therapy constituted 25 (15.3%). SLEDAI ≥ 10 accounted for 89 (54.6%) (Tables 1 and 2).

**Table 1:** Comparison of characteristics between cluster 1 and cluster 2 in SLE-PAH patients at baseline assessment

Characteristics	Overall (*n* = 163)	Cluster 1 (*n* = 134)	Cluster 2 (*n* = 29)	*P*-value*
**Demography**				
Female, *n* (%)	157 (96.3)	130 (97.0)	27 (93.1)	0.290
Median age at diagnosis (years)	37.0 (30.0, 49.0)	38.0 (30.0, 46.5)	33.0 (28.0, 50.5)	0.624
Duration (Mth)	24.0 (3.0, 84.0)	24.0 (3.0, 84.0)	24.0 (2.0, 78.0)	0.713
**Clinical features**				
Cutaneous lupus, *n* (%)	41 (25.5)	33 (24.6)	8 (27.6)	0.739
Oral or nasal ulcers, *n* (%)	15 (9.3)	12 (9.0)	3 (10.3)	0.732
Raynaud’s phenomenon, *n* (%)	43 (26.5)	37 (21.6)	7 (24.1)	0.702
Serositis, *n* (%)	100 (61.1)	77 (57.5)	23 (79.3)	**0.028**
Lupus nephritis, *n* (%)	69 (42.6)	49 (36.6)	21 (72.4)	** < 0.001**
Arthritis, *n* (%)	48 (29.0)	41 (30.6)	7 (20.4)	0.489
Alopecia, *n* (%)	22 (13.6)	16 (11.9)	6 (20.7)	0.233
Vasculitis, *n* (%)	7 (4.3)	6 (4.5)	1 (3.4)	1.000
Neuropsychiatric lupus, *n* (%)	11 (6.7)	8 (6.0)	3 (10.3)	0.415
Cardiac disorder, *n* (%)^a^	43 (26.4)	30 (22.4)	13 (44.8)	**0.013**
Mild ILD, *n* (%)^b^	25 (15.4)	21 (15.7)	4 (13.8)	1.000
APS,* n* (%)	5 (3.1)	5 (3.7)	0 (0.0)	0.587
Peripheral thrombosis, *n* (%)	7 (4.3)	3 (2.2)	4 (13.8)	**0.020**
Thrombocytopenia, *n* (%)^c^	41 (25.2)	30 (22.4)	11 (37.9)	0.080
Low complement, *n* (%)^d^	138 (84.7)	111 (82.8)	27 (93.1)	0.255
WHO FC III/IV, *n* (%)	113 (69.3)	90 (67.2)	23 (79.3)	0.198
SLEDAI-2 K ≥ 10, *n* (%)	89 (54.6)	67 (50.0)	22 (75.9)	**0.011**
6MWD (m), (IQR)	436.0 (282.0, 534.0)	446.0 (310.5, 535.0)	344.0 (123.0, 468.5)	**0.027**
**Laboratory findings**				
White blood cells (10^9^/L)	5.0 (3.7, 6.9)	4.9 (3.6, 6.8)	5.8 (4.0, 8.0)	0.157
Hemoglobin (g/L)	102.1 (91.5, 121.0)	104.5 (93, 123.1)	96 (75.5, 111.0)	**0.026**
Lymphocyte (10^9^/L)	1.0 (0.7, 1.6)	1.1 (0.8, 1.7)	0.9 (0.6, 1.1)	**0.008**
PDW (fL)	16.8 (16.4, 17.5)	16.8 (16.4, 17.5)	16.8 (16.1, 17.8)	0.854
D-dimer (mg/L)	0.4 (0.2, 0.9)	0.4 (0.2, 0.9)	0.7 (0.4, 1.8)	**0.014**
NT-proBNP (pg/ml)	3992.1 (1197.0, 12250.9)	2439.5 (913.8, 7357.7)	35000.0 (27201.7, 35000.0)	** < 0.001**
eGFR (ml/min/1.73m^2^)	107.9 (77.4, 120.2)	111.5 (93.8, 123.0)	35.4 (18.1, 82.4)	** < 0.001**
Creatinine (µmol/L)	58.0 (49.0, 79.0)	56.0 (47.9, 68.3)	155.0 (78.0, 297.5)	** < 0.001**
Serum uric acid (µmol/L)	341.0 (280.0, 466.0)	325.5 (258.5, 411.0)	572.0 (436.0, 611.0)	** < 0.001**
C3 (g/L)	0.6 (0.4, 0.9)	0.7 (0.5, 0.9)	0.4 (0.2, 0.8)	**0.002**
C4 (g/L)	0.1 (0.1, 0.2)	0.1 (0.1, 0.2)	0.08 (0.05, 0.2)	0.231
ESR (mm/h)	41.0 (15.0, 77.0)	41.0 (16.5, 77.8)	43.0 (7.2, 82.5)	0.449
CRP (mg/L)	6.0 (1.5, 21.6)	6.1 (1.5, 21.7)	4.8 (1.4, 18.9)	0.682
**Antibodies**				
ANA, *n* (%)	160 (98.2)	132 (98.5)	28 (96.6)	0.447
Anti-dsDNA antibody, *n* (%)	77 (47.2)	60 (44.8)	17 (58.6)	0.176
Anti-SSA antibody, *n* (%)	95 (59.4)	83 (61.9)	14 (48.3)	0.174
Anti-SSB antibody, *n* (%)	32 (19.6)	28 (20.9)	4 (13.8)	0.383
Anti-Ro52 antibody, *n* (%)	110 (67.5)	95 (70.9)	15 (51.7)	**0.046**
Anti-centromere antibody, *n* (%)	13 (8.0)	10 (7.5)	3 (10.3)	0.704
Anti-Scl-70 antibody, *n* (%)	6 (3.7)	4 (3.0)	2 (6.9)	0.290
Anti-nRNP/Sm antibody, *n* (%)	105 (64.8)	89 (66.4)	16 (55.2)	0.251
Anti-Sm antibody, *n* (%)	55 (33.7)	46 (34.3)	9 (31.0)	0.734
**TTE parameters**				
PASP (mmHg)	55.0 (45.0, 80.0)	55.0 (45.8, 80.3)	54.0 (44.5, 74.5)	0.690
TR (m/s)	3.4 (3.1, 4.1)	3.5 (3.1, 4.1)	3.3 (3.1, 4.0)	0.381
LVEF (%)	64.0 (61.0, 65.0)	64.0 (62.0, 65.0)	61.0 (54.0, 65.0)	**0.015**
**SLE treatment**				
GC alone, *n* (%)	55 (33.5)	43 (32.1)	12 (41.1)	0.337
HCQ, *n* (%)	128 (78.0)	108 (80.6)	20 (69.0)	0.167
IS				
MMF, *n* (%)	43 (26.4)	34 (25.4)	9 (31.0)	0.530
CYC, *n* (%)	39 (23.9)	36 (26.9)	3 (10.3)	0.059
GC + IS, *n* (%)	98 (59.7)	85 (63.4)	13 (44.8)	0.064
**PTDs, n (%)**	93 (57.0)	84 (62.7)	9 (31.0)	**0.002**
PDE-5I, *n* (%)	55 (33.7)	52 (38.8)	3 (10.3)	**0.003**
ERA, *n* (%)	65 (39.9)	58 (43.3)	7 (24.1)	0.056
PGI2, *n* (%)	18 (11.1)	15 (11.2)	3 (10.3)	1.000
Monotherapy, *n* (%)	68 (41.4)	60 (44.8)	8 (27.6)	0.089
Combination, *n* (%)	25 (15.2)	24 (17.9)	1 (3.4)	0.050

Results are expressed as median (interquartile ranger) or number (%). The categorical variables are compared by chi-squared, and Mann–Whitney *U* test was used for continuous variables

*Abbreviations: SLE* systemic lupus erythematosus, *PAH* pulmonary arterial hypertension, *ILD* interstitial lung disease, *APS* antiphospholipid syndrome, *WHO FC* World Health Organization Functional Class, *SLEDAI-2 K* systemic lupus erythematosus disease activity index-2000, *6MWD* 6-min walking distance, *PDW* platelet distribution width, *NT-proBNP* N-terminal pro-B-type natriuretic peptide, *eGFR* estimated glomerular filtration rate, *CRP* C-reactive protein, *ESR* erythrocyte sedimentation rate, *PASP* pulmonary artery systolic pressure, *TR* tricuspid regurgitation, *LVEF* left ventricular ejection fraction, *ANA* antinuclear antibodies, *dsDNA* double-stranded DNA, *RNP* ribonucleoprotein, *TTE* transthoracic echocardiography, *GC* glucocorticoids, *HCQ* hydroxychloroquine, *IS* immunosuppressants, *MMF* mycophenolate mofetil, *CYC* cyclophosphamide, *PTDs* PAH-targeted drugs, *PDE-5I* phosphodiesterase-5 inhibitor, *ERA* endothelin receptor antagonist, *PGI2* prostacyclin

^a^Cardiac disorders included pericarditis, myocarditis, cardiomyopathy, and valvulopathy

^b^Mild ILD determined by high-resolution computerized tomography

^c^Thrombocytopenia defined as platelets < 100 × 10^9^/L

^d^Low complement defined as C3 < 0.91 g/l or C4 < 0.14 g/l

^*^P-value < 0.05 is shown in bold

**Table 2:** Comparison of RHC parameters between cluster 1 and cluster 2 in SLE-PAH patients at baseline assessment

RHC parameters	Overall (*n* = 46)	Cluster 1 (*n* = 38)	Cluster 2 (*n* = 8)	*P*-value*
mPAP (mmHg)	40.0 (28.8, 49.3)	39.5 (28.0, 49.3)	42.5 (34.0, 51.3)	0.270
PAWP (mmHg)	5.0 (4.0, 7.0)	5.5 (4.0, 7.0)	4 (3.0, 8.8)	0.518
PVR (WU)	6.5 (4.6, 9.9)	6.4 (4.1, 9.4)	10.1 (5.6, 13.4)	**0.044**
CI (L/min × m^2^)	3.3 (2.5, 3.7)	3.4 (2.8, 3.7)	2.3 (2.1, 2.8)	**0.005**
RAP (mmHg)	3.0 (2.0, 3.0)	3.0 (2.0, 5.0)	3.5 (3.0, 6.5)	0.265
S_V_O_2_ (%)	67.5 (61.8, 72.9)	68.7 (63.5, 72.9)	64.3 (60.2, 73.4)	0.094

Results are expressed as median (interquartile ranger). Mann–Whitney *U* test was used for continuous variables

*Abbreviations: RHC* right heart catheterization, *mPAP* mean pulmonary arterial pressure, *PAWP* pulmonary arterial wedge pressure, *PVR* pulmonary vascular resistance, *WU* Wood units, *CI* cardiac index, *RAP* right arterial pressure, *S*_*V*_*O*_*2*_ mixed venous oxygen saturation

^*^P-value < 0.05 is shown in bold

### SLE-PAH patient survival

During the observation period, 28/163 (17.1%) patients died at a median of 27.0 months (IQR 7.3, 66.5) from diagnosis. The overall 1-, 3-, and 5-year survival rates in SLE-PAH patients were 93.9%, 87.2%, and 84.8%, correspondingly. Causes of death included 13 (46.4%) patients of heart failure, followed by 8 (28.6%) of respiratory failure, 5 (17.9%) of serious infection, 1 (3.6%) of cerebrovascular disease, and 1 (3.6%) of cirrhosis. For non-survivors, they had older age, shorter duration, higher platelet distribution width (PDW) level, higher ratio of WHO FC III/IV (100% vs. 63%), cardiac disorder (42.9% vs. 23.0%), and thrombocytopenia (42.9% vs. 21.9%) (*P* < 0.05). In the antinuclear antibody spectrum, the non-survivors’ anti-centromere antibody positivity was higher (21.4% vs. 5.2%), while the anti-dsDNA antibody was lower (28.6% vs. 51.1%) (*P* < 0.05). As for treatment, the proportion of hydroxychloroquine (HCQ) (57.1% vs. 83%) and MMF (7.1% vs. 30.4%) in the non-survivor group was lower (*P* < 0.05). Among RHC parameters and 6MWD, non-survivors had higher pulmonary vascular resistance (PVR) and cardiac index (CI) and shorter 6MWD (*P* < 0.05) (Supplementary Table S2, S3). There was no significant difference in SLEDAI-2 K, TTE parameters, and other regimens.

### Identification of subtypes in SLE-PAH

**Fig. 2:**
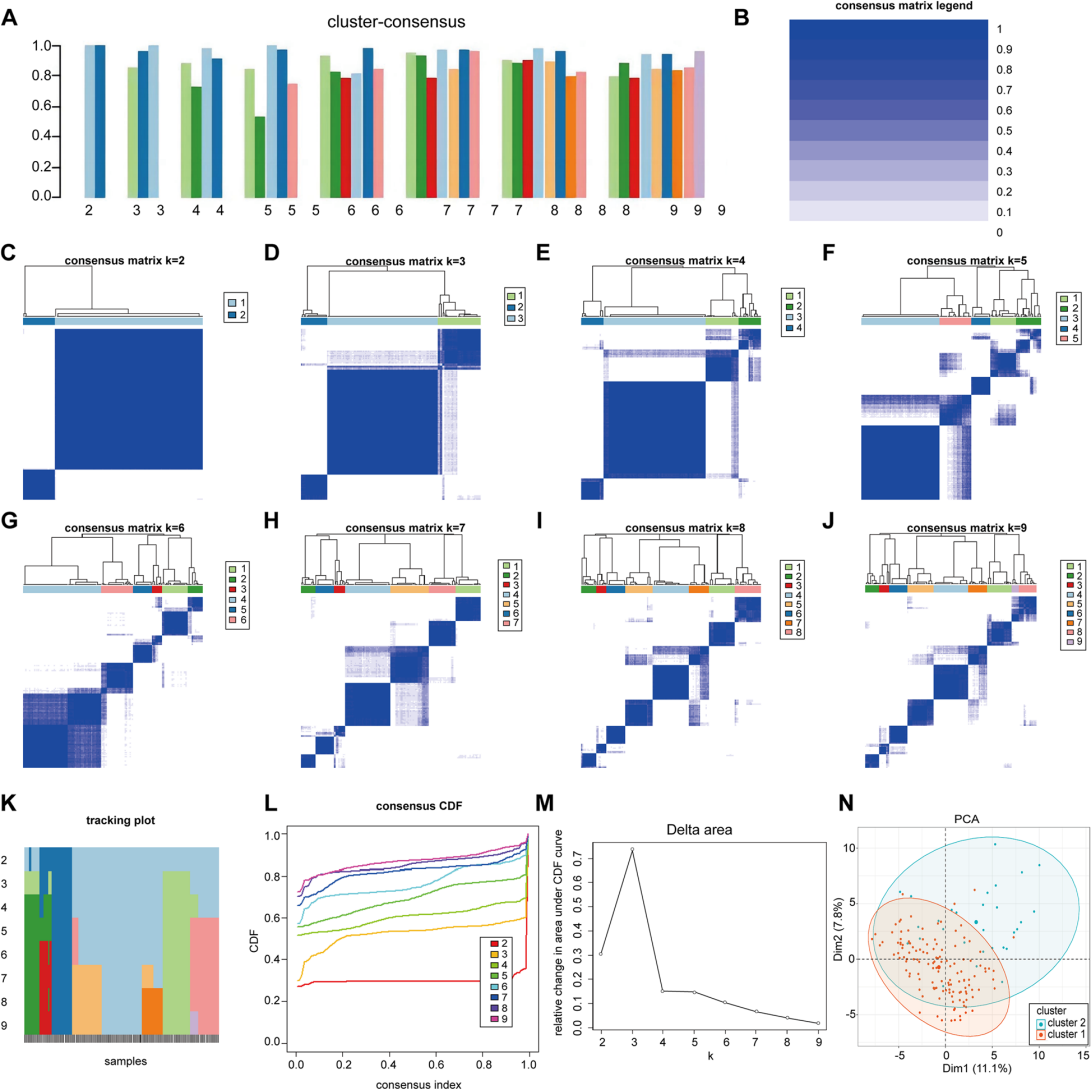
Identification of subtypes in SLE-PAH. **A** Consensus clustering score of clusters 2–9. **B** Consensus matrix legend of clusters 2–9. **C** Consensus clustering matrix when *k* = 2, **D ***k* = 3, **E ***k* = 4, **F ***k* = 5, **G ***k* = 6, **H ***k* = 7, **I ***k* = 8, and **J ***k* = 9. **K** Tracking plot of clustering. **L** CDF curves of clustering. **M** CDF delta area curves. **N** PCA visualizes the distribution of two subtypes. CDF, cumulative distribution function; PCA, principal component analysis

Consensus clustering was used to classify 163 SLE-PAH patients. Among 2–9 clusters, when the value was set to two (*k* = 2), the consensus score of each cluster was close to 1.0 (Fig. 2A). The consensus matrix from *k* = 2 to 9 was visualized in heatmaps (Fig. 2B–K). Fluctuation ranges of CDF curves were minimum at consensus index 0.2–0.4 when the cluster was set to two (*k* = 2) (Fig. 2L). The changes in delta areas were presented in CDF plots when *k* = 2–9 (Fig. 2M). In sum, 2 subtypes were selected as the optimal clustering, namely cluster 1 (*n* = 134) and cluster 2 (*n* = 29). PCA showed 2 subtypes had a distinguished discrimination (Fig. 2N).

**Fig. 3:**
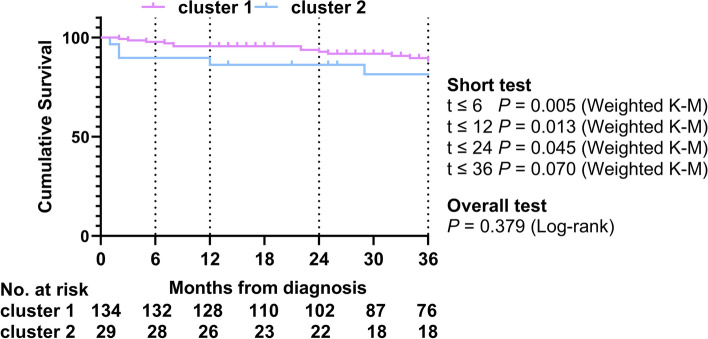
Kaplan–Meier survival curves of two clusters

In general, compared with cluster 1, SLE-PAH patients in cluster 2 had less favorable levels of poor cardiac, kidney, and coagulation function markers, with higher SLE disease activity, less frequency of PAH medications, and lower survival rate within 2 years (*P* < 0.05). These are described as follows:

Lupus nephritis (72.4% vs. 36.6%), serositis (79.3% vs. 57.5%), cardiac disorder (44.8% vs. 22.4%), peripheral thrombosis (13.8% vs. 2.2%), and high SLE disease activity (SLEDAI score ≥ 10) (75.9% vs. 50.0%) were more common in cluster 2 than in cluster 1 (*P* < 0.05). Among laboratory findings, the levels of creatinine, serum uric acid, D-dimer, and NT-proBNP in cluster 2 raised significantly, while the level of hemoglobin, lymphocyte, eGFR, and C3 declined (*P* < 0.05). However, there was no significant difference in erythrocyte sedimentation rate (ESR) and C-reactive protein (CRP). In the antinuclear antibody spectrum, less frequency of anti-Ro-52 antibody positivity occurred in cluster 2 (51.7% vs. 70.9%, *P* < 0.05). The ratio of PTDs in cluster 2 was lower (31.0% vs. 62.7%, *P* < 0.05), especially PDE-5Is (10.3% vs. 38.8%, *P* < 0.05), yet there was no statistical difference in other therapies. Regarding TTE, RHC parameters, and 6WMD, the cardiac function of cluster 2 was poorer, with lower left ventricular ejection fraction (LVEF) and cardiac index (CI), higher PVR, and shorter 6WMD (*P* < 0.05) (Tables 1 and 2, and S4). K-M analysis showed that the survival rate of cluster 2 was significantly poorer than cluster 1 within 2 years (86.2% vs. 92.8%, *P* < 0.05), whereas there was no prominent difference in survival rate over 2 years (Fig. 3).

### Comparison between SLE-PAH subtypes and IPAH

Forty-two IPAH patients were included for further comparison to SLE-PAH subtypes. In demography, there was a female predominance in cluster 1 compared with IPAH (97.0% vs. 83.3%, *P* < 0.05). Among clinical features, cluster 1 and cluster 2 both had a rate of serositis and renal involvement compared to IPAH (*P* < 0.05). In laboratory findings, hemoglobin and lymphocyte levels in both 2 clusters decreased, and D-dimer levels elevated (*P* < 0.05). As for detailed differences among the three groups, compared with IPAH, creatinine levels declined in cluster 1, while NT-proBNP, creatinine, and uric acid levels in cluster 2 raised (*P* < 0.05). Among TTE indicators, IPAH patients had higher PASP and tricuspid regurgitation (TR) than 2 clusters, but cluster 2 had lower LVEF (*P* < 0.05). In RHC, IPAH had higher mPAP and PVR and lower CI levels than cluster 1 (*P* < 0.05). In PTDs therapy, the percentage of PTDs (including PDE-5Is and ERAs) used in SLE-PAH subtypes occupied less than in the IPAH group (62.7% and 31.0% vs. 92.9%), especially less frequent in PTD combination therapy (*P* < 0.05). There was no significant difference in deaths among the three groups (Supplementary Table S5).

### Construction and validation of risk prediction model in SLE-PAH

**Fig. 4:**
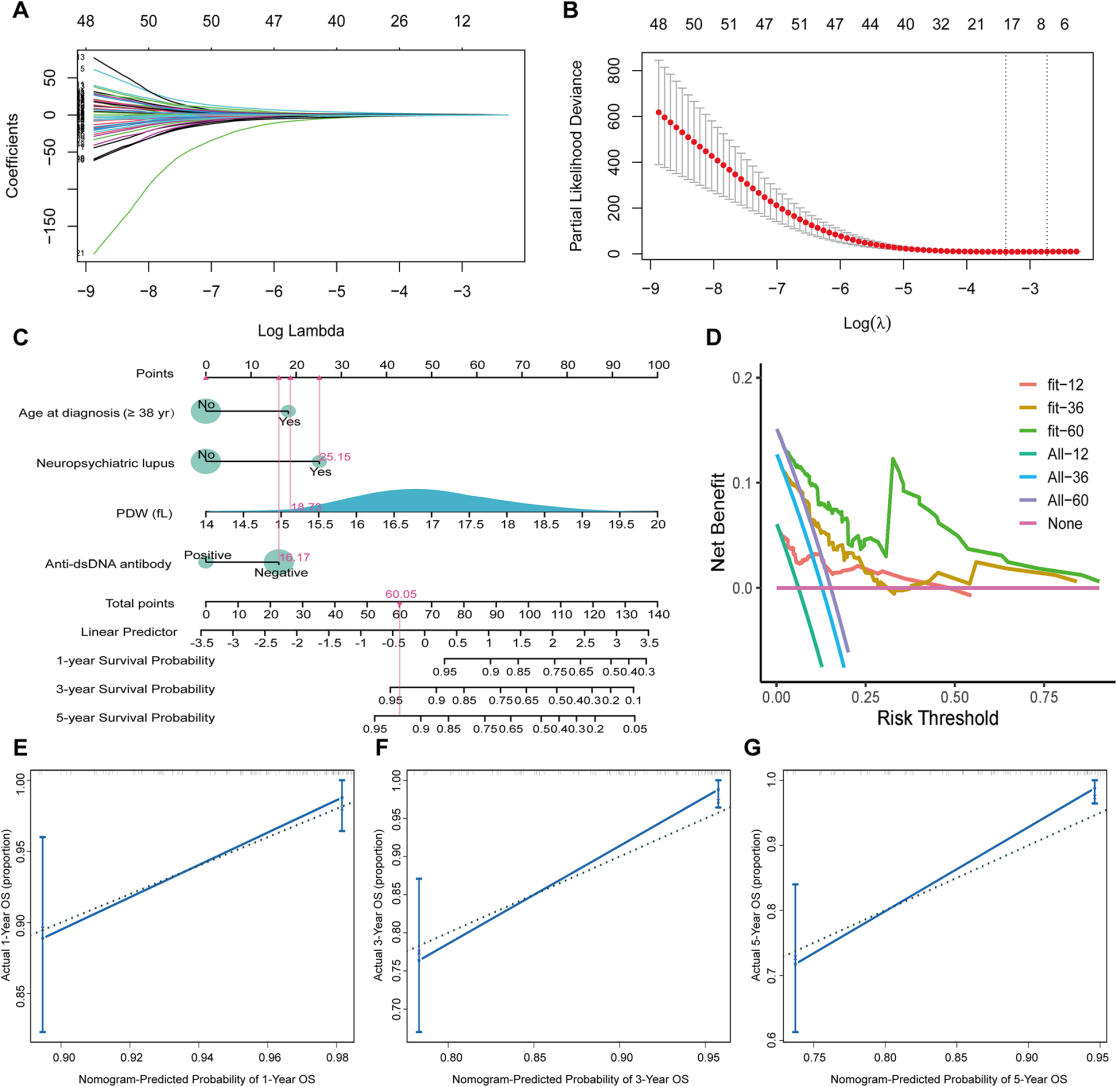
Construction and validation of risk prediction model of SLE-PAH. **A**, **B** Variable selection using the LASSO-Cox regression model. **C** Nomogram predicting the 1-, 3-, and 5-year survival probability of SLE-PAH patients. **D** DCA curves. **E**, **F** 1-, 3-, and 5-year calibration curves. LASSO, the least absolute shrinkage and selection operator; DCA, decision curve analysis; OS, overall survival

**Fig. 5:**
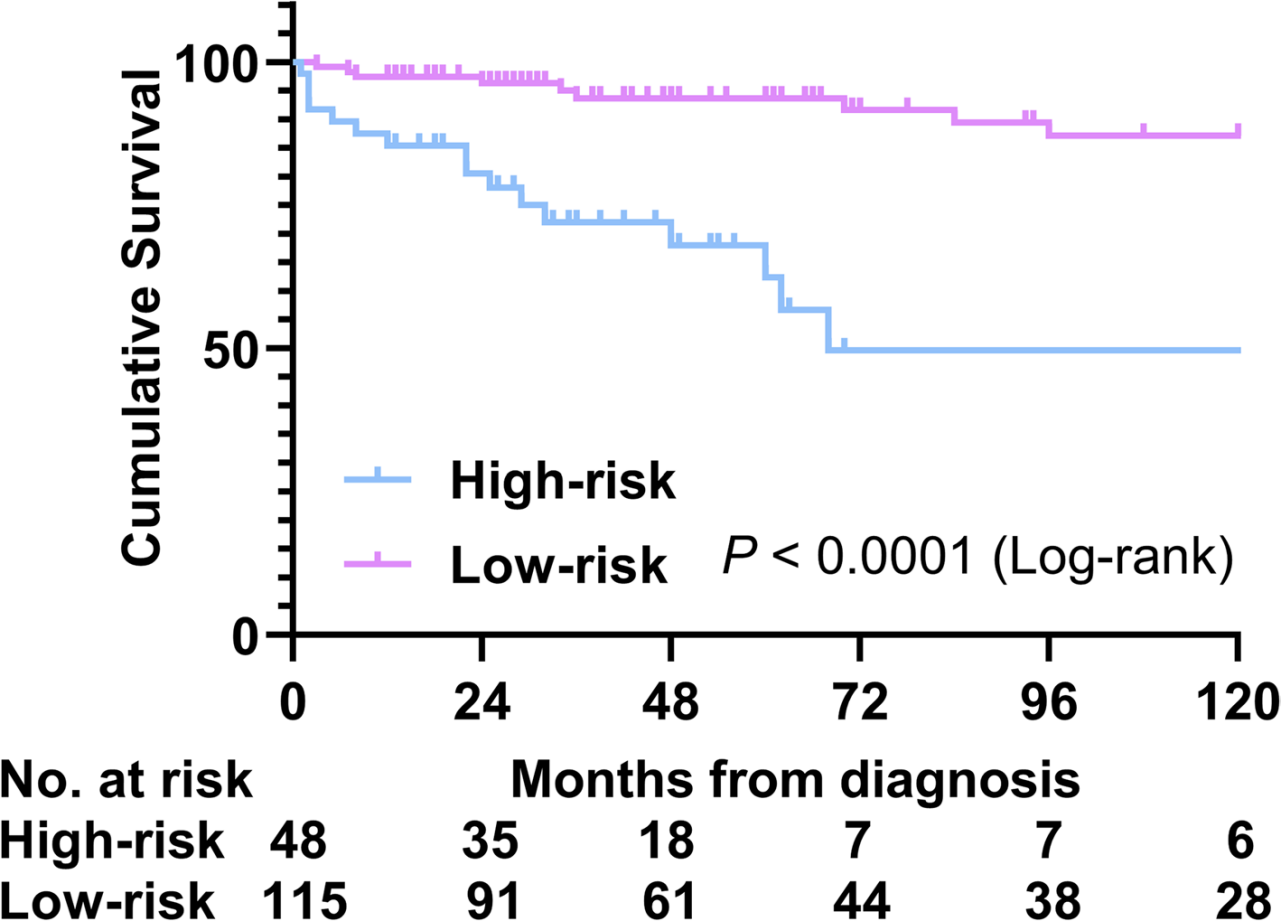
Kaplan–Meier survival curves of two risk groups

After LASSO-Cox regression analysis (Fig. 4A and B), four prognostic factors were finally identified: older age at diagnosis (≥ 38 years) [HR = 3.17, 95% CI (1.25–8.03)], anti-dsDNA antibody [HR = 0.28, 95% CI (0.11–0.75)], neuropsychiatric lupus [HR = 6.34, 95% CI (1.58–25.49)], and PDW [HR = 2.03, 95% CI (1.25–3.29)] (*P* < 0.05) (Table 3), of which anti-dsDNA antibody was protective and others were risk for death with SLE-PAH patients. Based on these factors, a nomogram model was constructed and evaluated, where the prognostic index = 0.922 × older age at diagnosis (≥ 38 years) − 0.816 × anti-dsDNA antibodies + 1.268 × neuropsychiatric lupus + 0.841 × PDW (Fig. 4C). The overall C-index of the model was 0.80, and after, the bootstrap correction was 0.77. The 1-, 3-, and 5-year C-index values were 84.4, 79.6, and 81.4, and after, the bootstrap correction were 77.0, 73.6, and 74.8, relatively. Additionally, the DCA curves and 1-, 3-, and 5-year calibration curves were visualized, indicating good clinical effectiveness and calibration (Fig. 4D–G). Furthermore, based on the optimal cutoff value of the risk score (risk score = 0.670), the K-M analysis showed that there was an eminent survival difference between high-risk and low-risk groups (*P* < 0.05) (Fig. 5).

**Table 3:** Prognostic factors associated with SLE-PAH patients (Cox proportional hazards model)

Factors	Univariate			Multivariate		
	HR	95% CI	*P*-value*	HR	95% CI	*P*-value*
Age at diagnosis (≥ 38 years)	2.54	1.11–5.81	**0.027**	3.17	1.25–8.03	**0.015**
Anti-centromere antibody	3.53	1.42–8.77	**0.006**	3.39	0.97–11.90	0.057
Anti-dsDNA antibody	0.43	0.19–0.98	**0.044**	0.28	0.11–0.75	**0.011**
Arthritis	0.54	0.21–1.44	0.219			
D-dimer (≥ 1.56 mg/L)	2.45	0.97–6.15	0.057	1.83	0.59–5.69	0.299
HCQ	0.35	0.16–0.74	**0.006**	0.56	0.23–1.35	0.197
Neuropsychiatric lupus	2.82	0.84–9.49	0.094	6.34	1.58–25.49	**0.009**
NT-proBNP	1.84	0.85–3.98	0.124			
PTDs	0.62	0.30–1.30	0.208			
Severe PASP (≥ 80 mmHg)	0.65	0.25–1.71	0.380			
PDW	2.23	1.43–3.46	0.000	2.03	1.25–3.29	**0.004**
6MWD	1.00	0.99–1.00	**0.001**	1.00	0.99–1.00	0.074
LVEF (< 55%)	0.36	0.05–2.70	0.319			
Thrombocytopenia	3.04	1.42–6.53	**0.004**	2.37	0.95–5.93	0.066
Thrombosis	3.31	0.99–11.02	0.052	2.39	0.55–10.27	0.243
Leukopenia	1.52	0.70–3.30	0.294			

Results are expressed as hazard ratio (95% confidence interval)

Age and D-dimer were categorized by survival ROC cutoff value. Thrombocytopenia was defined as platelets < 100 × 10^9^/L; leukopenia was defined as white blood cells < 3 × 10^9^/L

^*^P-value < 0.05 is shown in bold

## Discussion

As a life-threatening complication of SLE patients, PAH generally causes distinguished clinical worsens and poor outcomes. Thus, it is necessary to explore the clinical phenotype characteristics and prognostic factors in SLE-PAH. In this study, we identified two distinct subtypes of SLE-PAH based on unsupervised consensus clustering methodology for the first time and constructed a risk prediction model of SLE-PAH, with the ultimate goal of improving SLE-PAH assessment, treatment, and prognosis.

In our sample, the 1-, 3-, and 5-year overall survival rates were 93.9%, 87.2%, and 84.8%, respectively, which was higher than the data from a 2017 meta-analysis of 323 SLE-PAH patients (88%, 81%, 68%) and a large, multi-center Chinese cohort reported by the 2019 Chinese SLE Treatment and Research Group (CSTAR) study (92.1%, 84.8%, 72.9%) [4, 5]. The different ethnicity, cohort size, baseline characteristics, and treatment regimens across different regions and countries might be attributed to the heterogeneity in the long-term prognosis of SLE-PAH. In addition, heart failure was the leading cause of death in most SLE-PAH patients [26]. We found that 13 cases had heart failure among 28 non-survivors, which was in line with the early research. This might be because increasing PASP could cause a pronounced right ventricle afterload, eliciting progressive ventricular hypertrophy, right heart failure, and ultimately death [2].

SLE-PAH has complex pathogenesis and strong heterogeneity. Generally, there have been two putative pathological mechanisms of SLE-PAH, autoimmune-mediated inflammatory process and non-inflammatory vascular remodeling. As aforementioned, Sun et al. have divided 108 SLE-PAH patients into two subtypes, namely the vasculitic subtype and the vasculopathic subtype, trying to interpret these two mechanisms [11, 27]. To be specific, patients in the vasculitic subtype had systemic manifestations, high SLE disease activity, better response to drugs, and higher survival rates. In contrast, patients in the vasculopathic subtype were more likely to present purer PAH, that was, high pulmonary arterial pressure, poor cardiac function, poor response to drugs, and lower survival rate, albeit with mild inflammatory response and low disease activity. However, this classification of SLE-PAH has always been controversial.

In our study, we re-identified SLE-PAH subtypes through an integrative unsupervised consensus clustering and also found two distinct subtypes (cluster 1 and cluster 2). Compared with cluster 1, patients in cluster 2 had more organ involvement, higher disease activity, less PTD treatment, and poorer survival rate within 2 years. Furthermore, compared to IPAH, cluster 1 had female predominance and milder kidney and pulmonary damage, whereas cluster 2 had poorer cardiac and renal damage. Both clusters had a lower proportion of PTDs (PDE5-1I or ERA), especially less frequent in PTD combination therapy. In sum, two subtypes were different from the vasculitic and vasculopathic subtypes, suggesting diverse clinical phenotypes in SLE-PAH patients. It seems that inflammation and non-inflammation pathogenesis should not be completely isolated. Cross-interaction of both might lead to SLE-PAH progression. In a clinical view, when managing SLE-PAH patients, especially those who have cluster 2 characteristics, clinicians should focus on a 2-year treatment window and administer PTDs promptly for preventing irreversible pulmonary vascular damage.

Currently, the precise pathogenesis of SLE-PAH has not been fully elucidated. Several studies have explored the risk factors for death of SLE-PAH, such as poor cardiac function and exercise capacity; increased mPAP, PVR, BNP/NT-proBNP, and serum uric acid; and high rate of thrombocytopenia, pulmonary vasculitis, and Raynaud’s phenomenon [28–30]. In our study, similarly, we found PVR, the proportion of thrombocytopenia, and WHO FC III/IV were higher in non-survivors. In the risk prediction model, we newly identified four prognostic factors: older age at diagnosis (≥ 38 years), neuropsychiatric lupus, anti-dsDNA antibody, and PDW. As of today, Few studies have reported the relationship between neuropsychiatric lupus and PAH. Celfe et al. identified that neuropsychiatric lupus was more common in the PH group of SLE compared with the non-PH group [31]. Interestingly, in another study about neuropsychiatric SLE patients, Magro-Checa et al. showed that cardiovascular risk factors, especially arterial hypertension, were associated with ischemic changes in brain MRI, mainly lacunar stroke and brain atrophy [32]. Yet, the linkage between PAH and neuropsychiatric involvement in SLE needs to be further explored.

As for autoantibodies, conclusive evidence has not been obtained on whether lupus autoantibodies participated in SLE-PAH pathogenesis. Previous studies have indicated that the anti-RNP antibody in PAH might be implicated in injuring pulmonary vascular endothelial cells, inducing the proliferation of smooth muscle fibers [33, 34]. Antiphospholipid antibodies, especially the anticardiolipin antibody and lupus anticoagulant, were also related to increased risk for PAH occurrence [35, 36]. Anti-dsDNA antibodies are generally a hallmark of SLE diagnosis and classification, yet their role in PAH has not been fully understood. Studies have reported that anti-dsDNA antibodies, together with other autoantibodies, might directly damage vascular epithelial cells or form immune complexes depositing in the vascular wall, eliciting vasoconstriction, platelet aggregation, and thrombosis in SLE-PAH [37]. In the present study, however, we found that anti-dsDNA antibody was protective for death in SLE-PAH patients, which might reveal its complicated effect. As is known, anti-dsDNA antibody has different subclasses, including IgA, IgE, IgG, and IgM. However, not all of them contribute to tissue injuries in SLE. Ubiquitously, IgG and IgA correlate with SLE disease activity, but IgM was protective by inducing the eradication of apoptotic material and via immunomodulatory effects, thus attenuating cardiovascular dysfunction in SLE patients [38]. However, how anti-dsDNA subclasses exert a dual role in SLE-PAH courses is still unclear, and further research is needed to demonstrate.

Intriguingly, our results also confirmed that PDW was an independent risk factor for death in SLE-PAH. As an indicator of platelet activation, elevated PDW level represents a great dispersion in platelet volume and declined homogeneity [39]. As noted above, thrombocytopenia was strongly related to SLE-PAH prognosis. Previous studies have investigated that PDW rose significantly in IPAH and were positively associated with SLE disease activity [39, 40]. He et al. directly found that PDW could be a predictor of the early diagnosis of SLE-PAH [41]. These findings indicated that PDW which symbolizes abnormal platelet activity may play a pivotal role in SLE-PAH. After platelets were activated and destructed, the larger platelets with increased adhesion and aggregation would induce thrombogenesis, ascending PVR and PASP [42, 43]. Meanwhile, platelet activation could also stimulate inflammatory factors and complements releasing in SLE patients, damaging pulmonary vascular endothelial cells, promoting immune complexes depositing in the vessel wall, and eventually triggering the occurrence of pulmonary vascular diseases [44, 45].

There are several limitations to our study. Firstly, the retrospective nature and the selection of cases from a single center might have caused a selection bias. Given patients were selected from a center for SLE-PAH, more severe forms of the disease were recorded. Secondly, in our study, 88/205 individuals had underwent RHC estimation. Despite a previous study has defined a PASP of 40 mmHg measured by TTE as a good cutoff value for PAH diagnosis, RHC is the gold standard for PAH measurement. Finally, the sample size of this study is small, and the risk prediction model lacks external validation, so prospective and multicenter cohort studies are needed for further verification in the future.

## Conclusions

In this retrospective cohort study, we found two distinct subtypes in SLE-PAH patients based on consensus clustering analysis. Patients in cluster 2 had more organ involvement, higher SLE disease activity, and poorer survival rate within 2 years. Besides, a risk prediction model for the death of SLE-PAH patients was constructed, including older age at diagnosis (≥ 38 years), anti-dsDNA antibodies, neuropsychiatric lupus, and PDW. The model had great discrimination, calibration, and clinical practicability.

### Supplementary Information

**Additional file 1:** **Supplementary Table S1.** Cox proportional hazards assumption test of four prognostic variables. **Supplementary Table S2.** Comparison of characteristics between survivors and non-survivors in SLE-PAH patients at baseline assessment. **Supplementary Table S3.** Comparison of RHC parameters between survivors and non-survivors in SLE-PAH patients at baseline assessment. **Supplementary Table S4.** Comparison between cluster 1 and cluster 2 in SLE-PAH patients at baseline assessment. **Supplementary Table S5.** Comparison among IPAH, cluster 1 and cluster 2 in SLE-PAH patients at baseline assessment.

## Data Availability

The datasets used and/or analyzed during the current study are available from the corresponding author on reasonable request.

## Abbreviations

SLE: Systemic lupus erythematosus
PAH: Pulmonary arterial hypertension
ILD: Interstitial lung disease
APS: Antiphospholipid syndrome
WHO FC: World Health Organization Functional Class
SLEDAI-2K: Systemic lupus erythematosus disease activity index-2000
PDW: Platelet distribution width
NT-proBNP: N-terminal pro-B-type natriuretic peptide
eGFR: Estimated glomerular filtration rate
CRP: C-reactive protein
ESR: Erythrocyte sedimentation rate
PASP: Pulmonary artery systolic pressure
TR: Tricuspid regurgitation
LVEF: Left ventricular ejection fraction
ANA: Antinuclear antibodies
dsDNA: Double-stranded DNA
RNP: Ribonucleoprotein
TTE: Transthoracic echocardiography
GC: Glucocorticoids
IS: Immunosuppressants
HCQ: Hydroxychloroquine
MMF: Mycophenolate mofetil
CYC: Cyclophosphamide
PTDs: PAH-targeted drugs
PDE-5I: Phosphodiesterase-5 inhibitor
ERA: Endothelin receptor antagonist
PGI2: Prostacyclin
RHC: Right heart catheterization
mPAP: Mean pulmonary arterial pressure
PAWP: Pulmonary arterial wedge pressure
PVR: Pulmonary vascular resistance
WU: Wood units
CI: Cardiac index
RAP: Right arterial pressure
S_V_O_2_: Mixed venous oxygen saturation
6MWD: 6-Min walking distance
