# Supplementary material for: Clinical characteristics and prognosis in systemic lupus erythematosus-associated pulmonary arterial hypertension based on consensus clustering and risk prediction model
Source: Arthritis Res Ther. 2023 Aug 23;25:155. doi: 10.1186/s13075-023-03139-y (PMC10463535; doi:10.1186/s13075-023-03139-y)
Supplement: Supplementary file 1 — Additional file 1: Supplementary Table S1. Cox proportional hazards assumption test of four prognostic variables. Supplementary Table S2. Comparison of characteristics between survivors and non-survivors in SLE-PAH patients at baseline assessment. Supplementary Table S3. Comparison of RHC parameters between survivors and non-survivors in SLE-PAH patients at baseline assessment. Supplementary Table S4. Comparison between cluster 1 and cluster 2 in SLE-PAH patients at baseline assessment. Supplementary Table S5. Comparison among IPAH, cluster 1 and cluster 2 in SLE-PAH patients at baseline assessment. [file 13075_2023_3139_MOESM1_ESM.docx]

**Table S1**

Cox proportional hazards assumption test of four prognostic variables.

| Variables | Chisq | *P*-value |
| --- | --- | --- |
| Age at diagnosis (≥ 38 yr) | 3.058 | 0.08 |
| Anti-dsDNA antibody | 0.931 | 0.33 |
| Neuropsychiatric lupus | 1.271 | 0.26 |
| PDW | 1.719 | 0.19 |
| GLOBAL | 6.370 | 0.17 |

PDW, Platelet distribution width

**Table S2**

Comparison of characteristics between survivors and non-survivors in SLE-PAH patients at baseline assessment.

| Characteristics | Survivor (*n* = 135) | Non-survivor(*n* = 28) | *P*-value* |  |
| --- | --- | --- | --- | --- |
| **Demography** |  |  |  |  |
| Female, n (%) | 130(96.3) | 27(96.4) | 1.000 |  |
| Median age at diagnosis (yr) | 35.0(28.0, 46.0) | 45.0(36.3, 59.8) | **0.001** |  |
| Duration (Mth) | 24.0(4.0, 84.0) | 24.0(2.0, 93.0) | 0.916 |  |
| **Clinical features** |  |  |  |  |
| Cutaneous lupus, n (%) | 69(51.1) | 10(35.7) | 0.138 |  |
| Oral or nasal ulcers, n (%) | 15(11.1) | 0(0.0) | 0.076 |  |
| Raynaud's phenomenon, n (%) | 39(28.9) | 5(17.9) | 0.231 |  |
| Serositis, n (%) | 82(60.7) | 18(64.3) | 0.726 |  |
| Lupus nephritis, n (%) | 58(43.0) | 12(42.9) | 0.992 |  |
| Arthritis, n (%) | 43(31.9) | 5(17.9) | 0.139 |  |
| Alopecia, n (%) | 19(14.1) | 3(10.7) | 0.769 |  |
| Vasculitis, n (%) | 6(4.4) | 1(3.6) | 1.000 |  |
| Neuropsychiatric lupus, n (%) | 8(5.9) | 3(10.7) | 0.404 |  |
| Cardiac disorder, n (%)^a^ | 31(23.0) | 12(42.9) | **0.030** |  |
| Mild ILD, n (%)^b^ | 20(14.8) | 5(17.9) | 0.684 |  |
| APS, n (%) | 4(3.0) | 1(3.6) | 1.000 |  |
| Peripheral thrombosis, n (%) | 4(3.0) | 3(10.7) | 0.099 |  |
| Thrombocytopenia, n (%)^c^ | 29(21.5) | 12(42.9) | **0.018** |  |
| Low complement, n (%)^d^ | 113(83.7) | 25(89.3) | 0.456 |  |
| WHO FC Ⅲ/Ⅳ, n (%) | 85(63.0) | 28(100.0) | **<0.001** |  |
| SLEDAI-2K ≥ 10, n (%) | 75(55.6) | 14(50.0) | 0.591 |  |
| 6MWD (m) | 444.0(320.0, 535.0) | 270.0(135.0, 487.8) | **0.004** |  |
| **Laboratory findings** |  |  |  |  |
| White blood cells (10^9^/L) | 5.0(3.7, 6.9) | 4.8(3.4, 7.1) | 0.890 |  |
| Hemoglobin (g/L) | 104.0(93.0, 125.7) | 99.5(75.0, 109.6) | 0.023 |  |
| Lymphocyte (10^9^/L) | 1.0(0.7, 1.6) | 1.0(0.7, 1.5) | 0.548 |  |
| PDW (fL) | 17.3(16.7,17.9) | 18.0(17.3,18.6) | **0.011** |  |
| D-dimer (mg/L) | 0.4(0.2, 1.1) | 0.6(0.3, 0.9) | 0.542 |  |
| NT-proBNP (pg/ml) | 4057.2(1426.6, 11528.9) | 2990.9(654.2, 22022.78) | 0.535 |  |
| eGFR (ml/min per1.73m^2^) | 108.7(82.18,121.37) | 97.41(43.42,115.08) | 0.050 |  |
| Creatinine (µmol/L) | 58.0(49.0, 79.0) | 58.5(50.3, 111.0) | 0.353 |  |
| Serum uric acid (µmol/L) | 337.0(282.0, 461.0) | 357.5(237.8, 490.3) | 0.986 |  |
| C3 (g/L) | 0.7(0.4, 0.9) | 0.5(0.3, 0.7) | 0.110 |  |
| C4 (g/L) | 0.1(0.1, 0.2) | 0.1(0.1, 0.1) | 0.178 |  |
| ESR (mm/h) | 40.0(15.0, 75.0) | 62.5(13.5, 98.0) | 0.264 |  |
| CRP (mg/L) | 5.6(1.5, 19.7) | 10.0(1.5, 26.3) | 0.489 |  |
| **Antibodies** |  |  |  |  |
| ANA, n (%) | 132(97.8) | 28(100.0) | 0.426 |  |
| Anti-dsDNA antibody, n (%) | 69(51.1) | 8(28.6) | **0.030** |  |
| Anti-SSA antibody, n (%) | 79(58.5) | 18(64.3) | 0.572 |  |
| Anti-SSB antibody, n (%) | 26(19.3) | 6(21.4) | 0.793 |  |
| Anti-Ro52 antibody, n (%) | 91(67.4) | 19(67.9) | 0.963 |  |
| Anti-Centromere antibody, n (%) | 7(5.2) | 6(21.4) | **0.011** |  |
| Anti-Scl-70 antibody, n (%) | 6(4.4) | 0(0.0) | 0.591 |  |
| Anti-nRNP/Sm antibody, n (%) | 90(66.7) | 15(53.6) | 0.188 |  |
| Anti-Sm antibody, n (%) | 47(34.8) | 8(28.6) | 0.525 |  |
| **TTE parameters** |  |  |  |  |
| PASP (mmHg) | 81.0(55.0, 96.0) | 72.3(57.0, 85.2) | 0.836 |  |
| TR (m/s) | 4.1(3.4, 4.6) | 4.0(3.5, 4.3) | 0.977 |  |
| LVEF (%) | 64.0(62.0,65.0) | 63.0(60.3,64.0) | 0.178 |  |
| **SLE treatment** |  |  |  |  |
| GC alone, n (%) | 131(85.6) | 2(78.6) | 0.844 |  |
| HCQ, n (%) | 112(83.0) | 16(57.1) | **0.003** |  |
| IS |  |  |  |  |
| MMF, n (%) | 41(30.4) | 2(7.1) | **0.011** |  |
| CYC, n (%) | 34(25.2) | 5(17.9) | 0.408 |  |
| GC+IS, n (%) | 85(63.0) | 13(46.4) | 0.104 |  |
| **PTDs, n (%)** | 80(59.3) | 13(46.4) | 0.212 |  |
| PDE-5I, n (%) | 45(33.3) | 10(35.7) | 0.808 |  |
| ERA, n (%) | 58(43.0) | 7(25.0) | 0.077 |  |
| PGI2, n (%) | 14(10.4) | 4(14.3) | 0.547 |  |
| Monotherapy, n (%) | 86(63.7) | 13(46.4) | 0.121 |  |
| Combination, n (%) | 20(14.8) | 5(17.9) | 0.684 |  |

Results are expressed as median (interquartile ranger) or number(%). The categorical variables are compared by chi-squared, Mann-Whitney *U* test was used for continuous variables.

^a^Cardiac disorder included pericarditis, myocarditis, cardiomyopathy, and valvulopathy.

^b^Mild ILD determined by high resolution computerized tomography.

^c^Thrombocytopenia defined as platelets < 100×10^9^/L;

^d^Low complement defined as C3 < 0.91 g/l or C4 < 0.14 g/l;

*Abbreviations:* SLE, Systemic lupus erythematosus; PAH, Pulmonary arterial hypertension; ILD, Interstitial lung disease; APS, Antiphospholipid syndrome; WHO FC, World Health Organization Functional Class; SLEDAI-2K, Systemic lupus erythematosus disease activity index-2000; PDW, Platelet distribution width; NT-proBNP, N-terminal pro-B-type natriuretic peptide; eGFR, Estimated glomerular filtration rate; CRP, C-reactive protein; ESR, Erythrocyte sedimentation rate; PASP, Pulmonary artery systolic pressure; TR, Tricuspid regurgitation; LVEF, Left ventricular ejection fraction; ANA, antinuclear antibodies; dsDNA, double stranded DNA; RNP, Ribonucleoprotein; TTE, transthoracic echocardiography; GC, Glucocorticoids; HCQ, Hydroxychloroquine; IS, Immunosuppressants; MMF, Mycophenolate Mofetil; CYC, Cyclophosphamide; PTDs, PAH-targeted drugs; PDE-5I, Phosphodiesterase-5 inhibitor; ERA, Endothelin receptor antagonist; PGI2, Prostacyclin.

* *P*-value < 0.05 is shown in bold.

**Table S3**

Comparison of RHC parameters between survivors and non-survivors in SLE-PAH patients at baseline assessment.

| RHC parameters | Overall (*n* = 46) | Survivor(*n* = 37) | Non-survivor(*n* = 9) | *P*-value* |
| --- | --- | --- | --- | --- |
| mPAP (mmHg) | 40.0(28.8, 49.3) | 38.0(28.0, 49.5) | 45.0(39.0, 49.5) | 0.099 |
| PAWP (mmHg) | 5(4.0, 7.0) | 5(4.0, 7.0) | 5(3.0, 8.0) | 0.705 |
| PVR (WU) | 6.5(4.6, 9.9) | 6.4(4.1, 9.4) | 10.2(6.2, 13.4) | **0.034** |
| CI (L/min×m^2^) | 3.3(2.5, 3.7) | 3.3(2.7, 3.7) | 2.3(2.1, 3.2) | **0.044** |
| RAP (mmHg) | 3.0(2.0, 3.0) | 3.0(2.0, 5.0) | 5.0(2.5, 6.0) | 0.274 |
| S_V_O_2_ (%) | 67.5(61.8, 72.9) | 69.5(64.5, 72.9) | 61.8(59.5, 70.3) | 0.094 |

Results are expressed as median (interquartile ranger). Mann-Whitney *U* test was used for continuous variables.

*Abbreviations:* RHC, Right heart catheterization; mPAP, Mean pulmonary arterial pressure; PAWP, Pulmonary arterial wedge pressure; PVR, Pulmonary vascular resistance; WU, Wood units; CI, Cardiac index; RAP, Right arterial pressure; S_V_O_2_, Mixed venous oxygen saturation; 6MWD, 6-minute walking distance.

* *P*-value < 0.05 is shown in bold.

**Table S4**

Comparison between cluster 1 and cluster 2 in SLE-PAH patients at baseline assessment.

| Characteristics | cluster 1 (*n*=134) | cluster 2 (*n*=29) | *P*-value* |
| --- | --- | --- | --- |
| **Demography** |  |  |  |
| Female, n (%) | 130(97.0) | 27(93.1) | 0.290 |
| Median age at diagnosis (yr) | 38.0(30.0,46.5) | 33.0(28.0,50.5) | 0.624 |
| Duration (Mth) | 24.0(3.0,84.0) | 24.0(2.0,78.0) | 0.713 |
| **Clinical features** |  |  |  |
| Cutaneous lupus, n (%) | 33(24.6) | 8(27.6) | 0.739 |
| Oral or nasal ulcers, n (%) | 12(9.0) | 3(10.3) | 0.732 |
| Raynaud's phenomenon, n (%) | 37(21.6) | 7(24.1) | 0.702 |
| Serositis, n (%) | 77(57.5) | 23(79.3) | **0.028** |
| Lupus nephritis, n (%) | 49(36.6) | 21(72.4) | **<0.001** |
| Arthritis, n (%) | 41(30.6) | 7(20.4) | 0.489 |
| Alopecia, n (%) | 16(11.9) | 6(20.7) | 0.233 |
| Vasculitis, n (%) | 6(4.5) | 1(3.4) | 1.000 |
| Neuropsychiatric lupus, n (%) | 8(6.0) | 3(10.3) | 0.415 |
| Cardiac disorder, n (%)^a^ | 30(22.4) | 13(44.8) | **0.013** |
| Mild ILD, n (%)^b^ | 21(15.7) | 4(13.8) | 1.000 |
| APS, n (%) | 5(3.7) | 0 (0.0) | 0.587 |
| Peripheral thrombosis, n (%) | 3(2.2) | 4(13.8) | **0.020** |
| Thrombocytopenia, n (%)^c^ | 30(22.4) | 11(37.9) | 0.080 |
| Low complement, n (%)^d^ | 111(82.8) | 27(93.1) | 0.255 |
| WHO FC Ⅲ/Ⅳ, n (%) | 90(67.2) | 23(79.3) | 0.198 |
| SLEDAI-2K ≥ 10, n (%) | 67(50.0) | 22(75.9) | **0.011** |
| 6MWD (m), (IQR) | 446.0(310.5, 535.0) | 344.0(123.0, 468.5) | **0.027** |
| **Laboratory findings** |  |  |  |
| White blood cells (10^9^/L) | 4.9(3.6,6.8) | 5.8(4.0,8.0) | 0.157 |
| Hemoglobin (g/L) | 104.5(93,123.1) | 96(75.5,111.0) | **0.026** |
| Lymphocyte (10^9^/L) | 1.1(0.8,1.7) | 0.9(0.6,1.1) | **0.008** |
| PDW (fL) | 16.8(16.4,17.5) | 16.8(16.1,17.8) | 0.854 |
| D-dimer (mg/L) | 0.4(0.2,0.9) | 0.7(0.4,1.8) | **0.014** |
| NT-proBNP (pg/ml) | 2439.5(913.8,7357.7) | 35000.0(27201.7,35000.0) | **<0.001** |
| eGFR (ml/min per1.73m^2^) | 111.5(93.8,123.0) | 35.4(18.1,82.4) | **<0.001** |
| Creatinine (µmol/L) | 56.0(47.9,68.3) | 155.0(78.0,297.5) | **<0.001** |
| Serum uric acid (µmol/L) | 325.5 (258.5, 411.0) | 572.0(436.0,611.0) | **<0.001** |
| C3 (g/L) | 0.7(0.5,0.9) | 0.4(0.2,0.8) | **0.002** |
| C4 (g/L) | 0.1(0.1,0.2) | 0.08(0.05,0.2) | 0.231 |
| ESR (mm/h) | 41.0(16.5,77.8) | 43.0(7.2,82.5) | 0.449 |
| CRP (mg/L) | 6.1(1.5,21.7) | 4.8(1.4,18.9) | 0.682 |
| **Antibodies** |  |  |  |
| ANA, n (%) | 132(98.5) | 28(96.6) | 0.447 |
| Anti-dsDNA antibody, n (%) | 60(44.8) | 17(58.6) | 0.176 |
| Anti-SSA antibody, n (%) | 83(61.9) | 14(48.3) | 0.174 |
| Anti-SSB antibody, n (%) | 28(20.9) | 4(13.8) | 0.383 |
| Anti-Ro52 antibody, n (%) | 95(70.9) | 15(51.7) | **0.046** |
| Anti-Centromere antibody, n (%) | 10(7.5) | 3(10.3) | 0.704 |
| Anti-Scl-70 antibody, n (%) | 4(3.0) | 2(6.9) | 0.290 |
| Anti-nRNP/Sm antibody, n (%) | 89(66.4) | 16(55.2) | 0.251 |
| Anti-Sm antibody, n (%) | 46(34.3) | 9(31.0) | 0.734 |
| **TTE parameters** |  |  |  |
| PASP (mmHg) | 55.0(45.8,80.3) | 54.0(44.5,74.5) | 0.690 |
| TR (m/s) | 3.5(3.1,4.1) | 3.3(3.1,4.0) | 0.381 |
| LVEF (%) | 64.0(62.0,65.0) | 61.0(54.0,65.0) | **0.015** |
| RHC parameters^e^ |  |  |  |
| mPAP (mmHg) | 39.5(28.0,49.3) | 42.5(34.0,51.3) | 0.270 |
| PAWP (mmHg) | 5.5(4.0,7.0) | 4(3.0,8.8) | 0.518 |
| PVR (WU) | 6.4(4.1,9.4) | 10.1(5.6,13.4) | **0.044** |
| CI (L/min×m^2^) | 3.4(2.8,3.7) | 2.3(2.1,2.8) | **0.005** |
| RAP (mmHg) | 3.0(2.0,5.0) | 3.5(3.0,6.5) | 0.265 |
| S_V_O_2_ (%) | 68.7(63.5,72.9) | 64.3(60.2,73.4) | 0.094 |
| **SLE treatment** |  |  |  |
| GC alone, n (%) | 43(32.1) | 12(41.1) | 0.337 |
| HCQ, n (%) | 108(80.6) | 20(69.0) | 0.167 |
| IS |  |  |  |
| MMF, n (%) | 34(25.4) | 9(31.0) | 0.530 |
| CYC, n (%) | 36(26.9) | 3(10.3) | 0.059 |
| GC+IS, n (%) | 85(63.4) | 13(44.8) | 0.064 |
| **PTDs, n (%)** | 84(62.7) | 9(31.0) | **0.002** |
| PDE-5I, n (%) | 52(38.8) | 3(10.3) | **0.003** |
| ERA, n (%) | 58(43.3) | 7(24.1) | 0.056 |
| PGI2, n (%) | 15(11.2) | 3(10.3) | 1.000 |
| Monotherapy, n (%) | 60(44.8) | 8(27.6) | 0.089 |
| Combination, n (%) | 24(17.9) | 1(3.4) | 0.050 |

Results are expressed as median (interquartile ranger) or number (%). The categorical variables are compared by chi-squared, Mann-Whitney *U* test was used for continuous variables.

^a^Cardiac disorder included pericarditis, myocarditis, cardiomyopathy, and valvulopathy.

^b^Mild ILD determined by high resolution computerized tomography.

^c^Thrombocytopenia defined as platelets < 100×10^9^/L;

^d^Low complement defined as C3 < 0.91 g/l or C4 < 0.14 g/l;

^e^46

*Abbreviations:* SLE, Systemic lupus erythematosus; PAH, Pulmonary arterial hypertension; ILD, Interstitial lung disease; APS, Antiphospholipid syndrome; WHO FC, World Health Organization Functional Class; SLEDAI-2K, Systemic lupus erythematosus disease activity index-2000; 6MWD, 6-minute walking distance. PDW, Platelet distribution width; NT-proBNP, N-terminal pro-B-type natriuretic peptide; eGFR, Estimated glomerular filtration rate; CRP, C-reactive protein; ESR, Erythrocyte sedimentation rate; PASP, Pulmonary artery systolic pressure; TR, Tricuspid regurgitation; LVEF, Left ventricular ejection fraction; ANA, antinuclear antibodies; dsDNA, double stranded DNA, RNP, Ribonucleoprotein; TTE, Transthoracic echocardiography; GC, Glucocorticoids; IS, Immunosuppressants; HCQ, Hydroxychloroquine; MMF, Mycophenolate Mofetil; CYC, Cyclophosphamide; PTDs, PAH-targeted drugs; PDE-5I, Phosphodiesterase-5 inhibitor; ERA, Endothelin receptor antagonist; PGI2, Prostacyclin; RHC, Right heart catheterization; mPAP, Mean pulmonary arterial pressure; PAWP, Pulmonary arterial wedge pressure; PVR, Pulmonary vascular resistance; WU, Wood units; CI, Cardiac index; RAP, Right arterial pressure; S_V_O_2_, Mixed venous oxygen saturation;

* *P*-value < 0.05 is shown in bold.

**Table S5**

Comparison among IPAH, cluster 1 and cluster 2 in SLE-PAH patients at baseline assessment.

| Characteristics | IPAH (*n*=42) | cluster 1 (*n*=134) | cluster 2 (*n*=29) | *P*-value* |
| --- | --- | --- | --- | --- |
| **Demography** |  |  |  |  |
| Female, n (%) | 35(83.3) | 130(97.0)^#^ | 27(93.1) | 0.006 |
| Median age at diagnosis (yr) | 34.0(26.5,42.25) | 38.0(30.0,46.5) | 33.0(28.0,50.5) | 0.165 |
| Duration (Mth) | 12.0(3.0,36.0) | 24.0(3.0,84.0) | 24.0(2.0,78.0) | 0.294 |
| **Clinical features** |  |  |  |  |
| Serositis, n (%) | 1(2.4) | 77(57.5)^#^ | 23(79.3)^#^ | **<0.001** |
| Cardiac disorder, n (%) | 12(28.6) | 30(22.4) | 13(44.8) | **0.045** |
| Other pulmonary involvement, n (%) | 5(11.9) | 22(16.4) | 6(20.7) | 0.599 |
| Renal disorder, n (%) | 2(2.6) | 49(36.6)^#^ | 25(86.2)^#^ | **<0.001** |
| Thrombocytopenia, n (%) | 9(21.4) | 30(22.4) | 11(37.9) | 0.185 |
| WHO FC Ⅲ/Ⅳ, n (%) | 25(59.5) | 90(67.2) | 23(79.3) | 0.217 |
| 6MWD (m) | 400.5(344.8, 460.8) | 446.0(310.5, 535.0) | 344.0(123.0, 468.5) | **0.020** |
| **Laboratory findings** |  |  |  |  |
| White blood cells (10^9^/L) | 5.7(4.1,7.8) | 4.9(3.6,6.8) | 5.8(4.0,8.0) | 0.140 |
| Hemoglobin (g/L) | 127(107,151.3) | 104.5(93,123.1)^#^ | 96(75.5,111.0)^#^ | **<0.001** |
| Lymphocyte (10^9^/L) | 1.9(1.2,2.5) | 1.1(0.8,1.7)^#^ | 0.9(0.6,1.1)^#^ | **<0.001** |
| D-dimer (mg/L) | 0.2(0.1,0.4) | 0.4(0.2,0.9)^#^ | 0.7(0.4,1.8)^#^ | **<0.001** |
| NT-proBNP (pg/ml) | 2109.0(237.2,5152.4) | 2439.5(913.8,7357.7) | 35000.0(27201.7,35000.0)^#^ | **<0.001** |
| eGFR (ml/min per1.73m^2^) | 108.8(95.4,120.1) | 111.5(93.8,123.0) | 35.4(18.1,82.4)^#^ | **<0.001** |
| Creatinine (µmol/L) | 62.5(55.0,72.5) | 56.0(47.9,68.3)^#^ | 155.0(78.0,297.5)^#^ | **<0.001** |
| Serum uric acid (µmol/L) | 390.5 (252.2, 507.5) | 325.5 (258.5, 411.0) | 572.0(436.0,611.0)^#^ | **<0.001** |
| **TTE parameters** |  |  |  |  |
| PASP (mmHg) | 75.0(54.0,111.25) | 55.0(45.8,80.3)^#^ | 54.0(44.5,74.5)^#^ | **0.002** |
| TR (m/s) | 4.1(3.4,4.9) | 3.5(3.1,4.1)^#^ | 3.3(3.1,4.0)^#^ | **0.003** |
| LVEF (%) | 64.0(62.0,67.0) | 64.0(62.0,65.0) | 61.0(54.0,65.5)^#^ | **0.021** |
| **RHC parameters**^a^ |  |  |  |  |
| mPAP (mmHg) | 45.0(33.8,57.8) | 39.5(28.0,49.3)^#^ | 42.5(34.0,51.3) | **0.015** |
| PAWP (mmHg) | 7.0(4.3,9.0) | 5.5(4.0,7.0) | 4(3.0,8.8) | 0.147 |
| PVR (WU) | 8.3(5.5,14.2) | 6.4(4.1,9.4)^#^ | 10.1(5.6,13.4) | **0.019** |
| CI (L/min×m^2^) | 2.9(2.2,3.7) | 3.4(2.8,3.7)^#^ | 2.3(2.1,2.8) | **0.015** |
| RAP (mmHg) | 5.0(2.3,8.0) | 3.0(2.0,5.0) | 3.5(3.0,6.5) | 0.059 |
| S_V_O_2_, % | 63.7(57.3,69.0) | 68.7(63.5,72.9) | 64.3(60.2,73.4) | 0.035 |
| **PTDs, n (%)** | 39(92.9) | 84(62.7)^#^ | 9(31.0)^#^ | **0.000** |
| PDE-5I, n (%) | 31(73.8) | 52(38.8)^#^ | 3(10.3)^#^ | **<0.001** |
| ERA, n (%) | 30(71.4) | 58(43.3)^#^ | 7(24.1)^#^ | **<0.001** |
| PGI2, n (%) | 7(16.7) | 15(11.2) | 3(10.3) | 0.625 |
| Monotherapy, n (%) | 12(28.6) | 60(44.8) | 8(27.6) | 0.068 |
| Combination, n (%) | 27(64.3) | 24(17.9)^#^ | 1(3.4)^#^ | **<0.001** |
| **Outcome** |  |  |  |  |
| Death, n (%) | 8(19.0) | 21(15.7) | 7(24.1) | 0.532 |

Results are expressed as median (interquartile ranger) or number (%). The categorical variables are compared by chi-squared, Kruskal-Wallis test was used for continuous variables. Bonferroni was used to correct *P* value for Post hoc test

^a^88

^#^ adjusted *P-*value < 0.05 after Bonferroni correction, compared to IPAH

Thrombocytopenia defined as platelets < 100×10^9^/L; Other pulmonary involvement included interstitial lung disease, respiratory failure, pulmonary infection. Renal disorder included lupus nephritis, renal insufficiency, proteinuria.

*Abbreviations:* SLE, Systemic lupus erythematosus; PAH, Pulmonary arterial hypertension; WHO FC, World Health Organization Functional Class; NT-proBNP, N-terminal pro-B-type natriuretic peptide; eGFR, Estimated glomerular filtration rate; PASP, Pulmonary artery systolic pressure; TR, Tricuspid regurgitation; LVEF, Left ventricular ejection fraction; TTE, Transthoracic echocardiography; PTDs, PAH-targeted drugs; PDE-5I, Phosphodiesterase-5 inhibitor; ERA, Endothelin receptor antagonist; PGI2, Prostacyclin.

* *P*-value < 0.05 is shown in bold.
